# Ovarian Tumor Removed by Enucleation

**Published:** 1873-09

**Authors:** Walter Burnham

**Affiliations:** Lowell, Mass.


### Miscellaneous.

### Ovarion Tumour removed by Enucleation.

By WALTER BURNHAM, M. D., Lowell, Mass.

CASE CXCIX.—*Ovariotomy.*—On the 17th of last month, I was called to visit Miss A. W., of Vermont, aged 22, who has been suffering from the inconvenience of an enlargement of the abdomen for about four years. She had menstruated regularly, and had but little pain and inconvenience, except from the size and pressure. She could exercise freely, but not rapidly; and chose to be upon her feet a considerable portion of the time, as the sagging of the tumour relieved the pressure upon the lungs and heart.

I arrived there, in company with Dr. Sherwood, of Fairfield, at about ten (10) o'clock, A. M., and made a hasty examination, and soon was satisfied that I had a unilocular cyst only to contend with, and, also, that there were not extensive adhesions; but as I could only move it to a limited extent, I also concluded that it had only a short pedicle. I advised Dr. Sherwood that we had better make preparations and operate the same day. He at once sent a messenger for some other medical assistants, who were prompt at the appointed time. The table was prepared in the usual manner, and a tub placed under the table to receive the contents of the tumor, and at 1:30, P.M., she was dressed for the occasion and placed herself upon the table without assistance. Chloroform was administered by her attending physician till she was in an anæsthetic, state, when the napkin was changed for one moistened with ether, and immediately I was informed that she was ready for the operation. My first incision was made two inches below the umbilicus, and carried down over the linea alba about three inches to the peritoneum by a free cut; then I seized the peritoneum with the forceps, and carefully cut through it by a horizontal stroke of the scalpel, when I introduced a grooved director, and completed the incision through that membrane with a bistoury, as far as the angles of the incision through the skin. About two pints of serum flowed from this incision, which caused a little delay. I then introduced T. S. Wells's ovarian trochar into the cyst and drew off near fifty (50) pounds of clear, limpid serum, which passed into the tub under the table, through a three-fourths inch rubber tubing that was attached to the trochar. This done, I drew the sac through the incision upon the surface of the abdomen. I had previously informed Dr. Sherwood of my intention to enucleate the cyst in this case, and explained to him the process, that he might intelligently assist me if required.

I then made a small slit through the peritoneal coat near the pedicle, and with the handle of my scalpel separated the two coats from each other to a small extent, until I could grasp them in either hand, and at once concluded the separation by pulling them apart, and thus removed the entire sac proper as belonging to the tumor; while that portion composed of peritoneum was laid back upon the abdomen, that I might examine it, and wait a little for hæmorrhage to start, if at all. The effects of the atmosphere, though a high temperature, soon contracted and corrugated the peritoneum to less than half its size, when I separated the cyst from it. On examination of the inner surface of the peritoneum, I found the vessels spread out upon it in a complete net-work, like that of an inflamed conjunctiva largely magnified; but there was no hæmorrhage, except one small artery where I divided the peritoneal coat; and here a small clot had formed, and I thought best to put on a ligature, as I did also on one upon the omentum, leaving the ends out at the lower angle of the incision, to keep it open for the discharge of any matter that might be deposited in the cavity.

After waiting more than an hour to allow the force of the heart to return, the sac was covered by a warm napkin before returning it into the abdomen. But finding no bleeding, I then placed it back into the cavity of the abdomen, and closed the wound by three sutures, one of which I passed through the edge of the peritoneum where I made the slit to secure that point to the opening, in case any clot should form and require suppuration to remove it. Over this, adhesive straps and a compress of cotton, to fill the vacuum of the abdomen, were placed upon her, secured by a straight bandage.

I have made one hundred and ninety-nine (199) ovarian operations, and, so far as the removal of the tumor and completion of the operation was concerned, this was done sooner by six minutes than I have ever before performed it; and without any attempt at haste. But this method has the advantage also of not requiring any ligatures to bleeding vessels, or the pedicle, or any clamp even, for as there are no vessels cut there are none to bleed. But, as this was the first time I had made the operation in this manner, I thought it prudent to leave it so I could watch it myself for an hour.

I believe Dr. Miner, of Buffalo, N. Y., was the first to recommend this mode of treating the pedicle, and much credit is due to him for what seems to me a very great improvement over all others; both in convenience to the surgeon and in the safety of the patient.

After the delay in the dressings until the ether was fully exhausted, she was dressed for the bed and carefully laid into it, and the eighth of a grain of morphia given her. She soon after fell into a quiet sleep for an hour, and, waking, complained that she had had no dinner, and was hungry. Took a cup of gruel, which was repeated through the night as she demanded; took another eighth of a grain of morphia in six hours, and slept half the night.

18th, 6, A.M.—Had some headache; pulse had increased to 100; and, after inquiry, I found she had suffered for two hours with retention of urine. I drew off about a quart of water, which relieved her head and the pulse returned to 80.

May 31, received a letter stating that she had been doing well all the time, had good appetite, etc. She is now nearly well, the sutures removed, the ligatures have both come away, and the wound all closed; but I do not think it best to allow her to go out too soon, or to move about the house much.

I shall be glad to see other surgeons adopting Dr. Miner's plan by enucleation, and let the profession know the results.

I consider this case as cured in two weeks, although it is prudent to require her to remain quiet for several weeks more. I have never closed the wound or allowed it to close, until all the ligatures have come away; and I have many times seen a great advantage in having the ends to manipulate with where abscess has formed, and I have never yet seen any objection to their presence. Besides I have several times known serious harm, and even fatal results, where other surgeons have cut the ligatures short and closed the abdomen over them. Abscesses have formed and opened in the perineum by the rectum, vagina, and at the point of incision. In all of my cases, I have had but one where abscess or suppuration has followed the operation with bad results. That one occurred twenty (20) days after, and rapidly terminated fatally, and it was not till after a *post-mortem* examination that an abscess was suspected, so rapid was the progress.—*Boston Med. and Surg. Journal.*

